# Values of Integrated Care: A Systematic Review

**DOI:** 10.5334/ijic.4172

**Published:** 2018-11-15

**Authors:** Nick Zonneveld, Naomi Driessen, René A. J. Stüssgen, Mirella M. N. Minkman

**Affiliations:** 1 TIAS School for Business and Society/Tilburg University, NL; 2 Vilans, National Centre of Excellence in Long Term Care, NL; 3 Nivel, Netherlands Institute for Health Services Research, NL

**Keywords:** integrated care, values, collaboration, behaviour, governance

## Abstract

**Introduction:**

Although substantial generic knowledge about integrated care has been developed, better understanding of the factors that drive behaviour, decision-making, collaboration and governance processes in integrated care networks is needed to take integrated care forward. To gain more insight into these topics and to understand integrated care in more depth, a set of underlying values of integrated care has been developed and defined in this study.

**Theory and methods:**

A systematic literature review was conducted to identify the underlying values of integrated care. Values theory was used as a theoretical framework for the analysis.

**Results:**

This study identified 23 values in the current body of knowledge. The most frequently identified values are ‘collaborative’, ‘co-ordinated’, ‘transparent’, ‘empowering’, ‘comprehensive’, ‘co-produced’ and ‘shared responsibility and accountability’.

**Discussion and conclusion:**

The set of values is presented as a potential basis for a values-driven approach to integrated care. This approach enables better understanding of the behaviours and collaboration in integrated care and may also be used to develop guidance or governance in this area. The practical application of the values and their use at multiple levels is discussed. The consequences of different stakeholder perceptions on the values is explored and an agenda for future research is proposed.

## Introduction

Integrated care is often proposed as future direction for the development of healthcare systems in many countries. Because people are living longer with more chronic conditions, the number of people with multiple health and social care needs is growing [1]. To meet these complex needs, knowledge and skills are required which span multiple disciplines in various sectors, for instance primary care, long-term care and social care. These developments put pressure on the delivery, management and funding of care services. In order to address this and to improve quality of care and quality of lives, integrated care is often introduced as a leading paradigm. The World Health Organisation (WHO), for instance, acknowledges the importance of integrated care in its vision and global strategy for health services delivery [2], and there is a proliferation of integrated care initiatives in many different countries, settings and environments [3,4,5]. The WHO defines integrated care (or integrated health services delivery) as “an approach to strengthen people-centred health systems through the promotion of the comprehensive delivery of quality services across the life-course, designed according to the multidimensional needs of the population and the individual and delivered by a coordinated multidisciplinary team of providers working across settings and levels of care. It should be effectively managed to ensure optimal outcomes and the appropriate use of resources based on the best available evidence, with feedback loops to continuously improve performance and to tackle upstream causes of ill health and to promote well-being through intersectoral and multisectoral actions” [6, p. 10]. Furthermore, integrated care aims to contribute towards improving population health, improving individual experiences of care, and reducing costs of care per capita, also known as the Triple Aim objectives [7].

While the objective is promising, integrated care remains a complex phenomenon which takes place at multiple levels, with various interventions, stakeholders and contextual factors that can influence processes and results [8,9,10,11]. The evidence for positive outcomes in integrated care is mixed for various reasons [12,13,14,15,16]. Furthermore, many different definitions of integrated care are used in scientific literature and in practice [2,6,17,18,19,20,21]. The literature describes numerous concepts similar to integrated care, e.g. ‘care coordination’, ‘collaborative care’ or ‘comprehensive care’. These definitions and concepts overlap to some extent, however the description of integrated care is not uniform and is still ambiguous.

Various efforts to analyse the complexity and provide a framework for the concept of integrated care, have led to the development of a substantial body of generic knowledge in recent years. As well as defining integrated care, several studies describe integrated care interventions, sets of measurements and generic ingredients [8,10,22,23,24,25,26,27,28]. Some of these studies resulted in conceptual models and frameworks, and although the focus may be different, they contain many common factors; the descriptions of which are similar and may overlap. For instance, the role of inter-professional collaboration within and between organisations is reflected in multiple models [14,23,24]. This generic knowledge is applicable in a broad range of integrated care settings. In practice, however, knowledge is tailored to local needs and circumstances, resulting in a variety of ways in which integrated care is executed.

Current knowledge about integrated care provides the basis for the development of integrated care initiatives, but to take integrated care a step further, deeper understanding of collaboration and behaviour in integrated care is needed. Since integrated care is a collective process, its implementation and execution depends on collaboration between individuals and organisations, such as clients, their families, professionals, governments and health insurers. Although working together, these actors may have different views, interests and objectives [29]. The identification of values in integrated care can therefore provide more insight into what drives the behaviours and decision-making of the various actors involved in integrated care processes [30,31]. What values are considered to be important and which ones influence behaviours, decision-making and perception of quality? This study aims to identify the underlying values of integrated care described in the current literature.

### Value and values in integrated care

The concepts of ‘value’ and ‘values’ are being mentioned more frequently in health- and integrated care literature and practice, mostly in relation to defining quality, guiding professional behaviour and aligning collaboration. When interpreting these concepts, it is important to be aware that *value* and *values* are used as different concepts with different meanings. This study focuses on *values*, which can be defined as meaningful beliefs, principles or standards of behaviour, referring to desirable goals that motivate action [30,31]. The term *value*, used in for instance Value-based healthcare, refers to the degree of success shown by a provider in meeting the needs of clients, relative to costs [32,33,34].

When looking at *values*, the healthcare sector has a tradition of professional and ethical codes for professionals that prescribe values, principles and quality standards in relation to professional behaviour [35]. Professional codes facilitate and guide professionals in their daily work and form a template for professional decision-making and behaviour [35,36]. In addition to professional codes, we also see codes which are used to guide organisations. An example is the Governance Code for healthcare providers in the Netherlands which states that care providers should define their values to determine their role in society. Besides common values, such as integrity, transparency and efficiency, every care provider should define their own individual values, corresponding to their specific position and objectives [37].

The importance of values is also acknowledged in integrated care literature and practice. Values are regarded as essential for increasing staff commitment to delivering the best quality for clients in successful integrated care practices [38]. Shared values across professionals and organisations are considered to be important factors in informal coordination and collaboration processes [39]. Furthermore, better understanding of the values of integrated care is necessary for the delivery of improved quality of care and client experiences [40].

Despite the growing attention being paid to values in the literature and practice, there is a lack of information about the actual relevant values in integrated care and their definition. The World Health Organisation recently published a strategic report that addresses values: ‘Global strategy on people-centred and integrated health services’ (interim report) [2]. In this report it is stated that the different approaches in people-centred and integrated health service delivery “should be grounded in a common set of principles. These provide a unifying values framework” [2, p. 11]. This report contains a first set of guiding principles of integrated care, developed in collaboration with the International Foundation for Integrated Care [2,41]. This set of principles was developed by considering the views of the partners involved in the development of the interim report, but the findings have not yet been systematically assessed.

### Study aims and objectives

Identification of the underlying values of integrated care enables better and deeper understanding of collaboration and behaviour in integrated care, and could also help to define quality in integrated care. The theoretical contribution of this study is to identify the values of integrated care from a systematic review of the current literature. The research question posed in this study is: based on current literature, what values underpin integrated care, and how can these values be described?

## Theory and methods

### Theoretical background

To identify the values which are associated with integrated care, it is necessary to have a deeper understanding of values as a theoretical concept. Many social scientists have been working on values in recent decades. In the 1950s Kluckhohn referred to values as conceptions of the desirable [42] that influence actions, distinguish individuals and characterise groups [30]. Later, in the 1970s, Rokeach added that values give meaning to these actions and behaviours. He considered values to be the beliefs that specific end-states or modes are preferable to an opposite situation [30]. In sociological theory, values are seen as moral compasses, determining what is important in our lives [43]. Our values form the core of our identity [44] and our behaviour can be a manifestation of our values [45]. The role of values in organisational processes has also been studied [46]. Rokeach distinguishes so-called supra-individual values – societal, institutional and organisational values – and discusses the relationships between these types of values [47]. Since integrated care is about coordination across professionals, providers, settings and levels of care [48] and other societal and organisational processes, this supra-individual perspective could also be relevant for this study.

While classical theorists such as Kluckhohn and Rokeach contributed to the conceptualisation of values, Schwartz contributed to the applicability and measurability of values in modern times. He drew on the work of Kluckhohn and Rokeach by developing a ‘Theory of Basic Values’ [31] and testing values empirically in many different countries [49]. In the Schwartz Value Survey (SVS) he relates values explicitly to guiding principles, asking respondents to rate the importance of values as a “guiding principle” in their lives on a 9-point scale [49]. Schwartz and Bilsky [50] also introduced five common characteristics of values, based on the body of knowledge at that time:

- values are concepts or beliefs.
- values refer to desirable goals, end states or behaviours.
- values transcend specific situations and objects. E.g. contrary to attitudes, values remain relevant in multiple contexts: in personal relationships, in work or in politics.
- values are the guiding principles in life and, as such, are used in the selection or evaluation of events, policies or behaviour. They serve as criteria for the preferred course of action.
- values are ordered by relative importance.

Individuals and groups of people vary in the importance they attach to particular values. In other words, they have different value hierarchies. In this way, values characterise individuals, groups and organisations, and can be used in the interpretation of their actions, behaviours and attitudes [30,31]. For example: people who attach more importance to freedom and flexibility than to housing security, would rather rent a house than buy one. Values can also be used to elucidate organisational behaviour and decision-making. For instance, the relative importance of the values ‘safety’ and ‘privacy’ may influence decisions about video surveillance in elderly care homes. If privacy prevails, the elderly care home may opt for an alternative solution.

Values theory may also be relevant in an integrated care setting, where decision-making processes and collaboration between multiple stakeholders with varying interests play a role [29]. By the absence of formal hierarchy in more horizontal collaboration, other processes of decision-making occur in which values could play a role. The aim of this study is to systematically identify underlying values of integrated care in the current literature, guided by the theoretical foundations described above. The search terms used in the systematic review were derived from Schwartz’ publications [31,49,50], and the content analysis was guided by the insights of Schwartz and Bilsky [50]. In this study, concepts or text fragments introduced in the literature are only considered to be values when they meet Schwartz and Bilsky’s five common characteristics of values [50]. Lastly, the insights of Hitlin and Piliavin [30], Schwartz [31], Rokeach [47] were used in the discussion section to reflect on the findings.

### Methods

#### Systematic review of the literature

The main objective of this study is to identify the values underpinning integrated care described in the current literature. A systematic electronic database search was conducted, in which we focused on papers in peer-reviewed journals. We searched the PubMed, PsycInfo and EBSCO Medline databases from 2006 to July 11^th^ 2017. Articles and reports were also retrieved by tracking reference lists. Empirical, theoretical and conceptual articles, written in English, were included in the search strategy (see Appendix 1).

We searched for references that had the terms ‘values’, ‘principles’ or similar nomenclature [31,50] in the title, combined with frequently used definitions and terminologies of integrated care, such as ‘coordinated care’, ‘person centred integrated care’ and ‘care coordination’ [18]. Some articles contain conceptual models that describe the building blocks or key elements of integrated care. These models may include certain values [31] and since they also support theory building, we also searched for articles with the terms ‘models’ and ‘frameworks’ in the title and abstract.

All search results were imported in MS Excel and duplicates were removed. Articles that contained definitions or descriptions of underlying values that fitted with Schwartz and Bilsky’s five common characteristics of values [50] were eligible for inclusion in the literature review. Two researchers independently screened the titles and abstracts of the search results. Disagreements were resolved by consensus discussions. If consensus could not be reached, a third researcher was consulted. The full-text articles were also independently assessed by two researchers. As before, any disagreements were resolved by discussion and when consensus could not be reached, a third researcher was consulted.

#### Data extraction and content analysis

Two data types were extracted from the articles included in the review. First, of each article the author(s), year of publication, title, country, scope of the article, study design and perspective were noted (see Appendix 2). Thereafter, all included articles were subject to directed content analysis [51].

All manuscripts were imported into MaxQDA software, and were independently reviewed by two researchers. Text fragments that contained all five features of values described by Schwartz and Bilsky [50] were highlighted. All highlighted text was coded using a predetermined coding sheet wherever possible. The predetermined coding sheet was based on the set of 16 principles presented in the article ‘Principles of Integrated Care’ by Ferrer and Goodwin (2014). This set was chosen for the following reasons: 1) in his publications, Schwartz operationalises values as “guiding principles in life” [31,49,50]. This set specifically refers to guiding principles in integrated care, 2) Ferrer and Goodwin’s principles were identified by consideration of the views and comments from international stakeholders and partners in the field of integrated care, and 3) the principles have been used in the policy report ‘Global Strategy on People-Centred and Integrated Health Services’ published by the World Health Organisation [2]. Text fragments that could not be coded into one of the predetermined categories were coded with a new label that summarised the essence of the text fragment. The coding sheet was only extended with any new labels that emerged during the analysis if 1) both researchers agreed that the particular value was an addition to the existing coding sheet, and 2) the particular value met all five characteristics of values described by Schwartz and Bilsky [50]. Subsequently, all coded text fragments were categorised and sorted per value label. Values supported by less than three articles were excluded. Any differences or disagreements to arise from the simultaneous coding were resolved by discussion between the two researchers. If no consensus was reached, a third researcher was consulted.

In the next step the values identified were described. First, all coded text fragments were sorted per value. Subsequently, two researchers independently divided the text fragments into segments representing the characteristics of the corresponding value. Thereafter, the characteristics of the values were discussed by the two researchers, resulting in a list of characteristics per value. Lastly, the lists of characteristics were processed into separate value descriptions. The descriptions of the 16 predetermined categories were developed by enhancing their initial descriptions [41] with the additional characteristics from the analysis. The complete analysis process was supervised by a fourth researcher.

## Results

**Figure 1:**
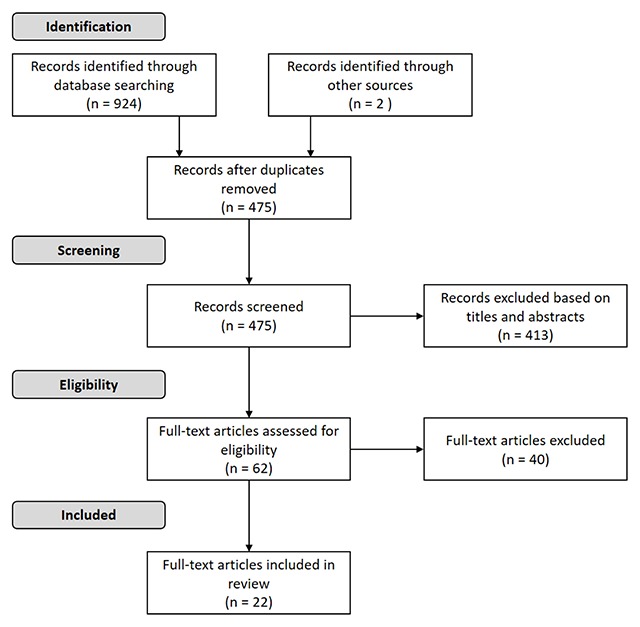
PRISMA flow diagram showing the study selection process.

A total of 924 records were identified by searching the databases. Two records were added by tracking reference lists. After removing duplicates, 475 articles remained. After screening titles and abstracts, 62 full-text articles were assessed for eligibility. Out of these 62 articles, 40 articles were excluded. The reasons for exclusion were recorded, e.g. articles that describe models for implementation processes, but no substantive elements of integrated care. Finally a total of 22 publications were retained for content analysis (see the PRISMA flow diagram in Figure 1).

The characteristics of the included full-texts are described in Table 1. Approximately 73% of the included articles originated from North America and 27% from Europe. The included articles are mostly qualitative descriptive papers (N = 18), and most of the included full-texts are written from a researcher’s or expert’s perspective (N = 18). Appendix 2 provides a list of the included full-texts.

**Table 1:** Characteristics of the full-text articles (N = 22).

	Full-texts (N = 22)	In %

**Countries of origin**		

United States of America	11	50.0
Canada	5	22.7
The Netherlands	2	9.1
Belgium	1	4.5
England	1	4.5
Italy	1	4.5
Sweden	1	4.5
**Study designs**		

Qualitative: descriptive	18	81.8
Systematic review	3	13.6
Mixed methods: embedded design	1	4.5
**Perspectives**		

Researcher/expert	18	81.8
Professional	3	13.6
Client	1	4.5

Content analysis of the 22 articles resulted in 23 values and their corresponding descriptions (see Table 2). The 23 values identified have been generally described and can be applied to a broad range of circumstances. The values that were identified most frequently are ‘collaborative’ (N = 20), ‘co-ordinated’ (N = 19), ‘transparent’ (N = 15), ‘empowering’ (N = 13), ‘comprehensive’ (N = 13), ‘co-produced’ (N = 13) and ‘shared responsibility and accountability’ (N = 13). The values ‘innovative’ (N = 4), ‘trustful’ (N = 4), ‘proficient’ (N = 3) and ‘safe’ (N = 3) were reported least of all.

**Table 2:** Values, descriptions and references.

#	Value label	Description	References

1	**Collaborative**	Professionals work together in teams, in collaboration with clients, their families and communities, establishing and maintaining good (working) relationships.	[39,41,52,53,54,55,56,57,58,59,60,61,62,63,64,65,66,67,68,69] (N = 20)
2	**Co-ordinated**	Connection and alignment between the involved actors and elements in the care chain, matching the needs of the unique person. Between professionals, clients and/or families, within teams and across teams.	[39,41,52,53,54,55,58,59,60,61,63,64,65,66,67,68,69,70,71] (N = 19)
3	**Transparent**	Openly and honestly giving insight in information, decisions, consequences and results, between clients, their families, professionals and providers.	[52,54,57,58,59,61,62,63,64,65,66,67,68,69,71] (N = 15)
4	**Empowering**	Facilitating and supporting people to build on their strengths, make their own decisions, manage their own health and take responsibility for it.	[39,41,53,55,57,59,60,61,63,64,66,67,68] (N = 13)
5	**Comprehensive**	The availability of a wide range of services, tailored to the evolving needs and preferences of clients and their families.	[39,41,52,53,54,58,59,60,63,64,67,68,70] (N = 13)
6	**Co-produced**	Engaging clients, their families and communities in the design, implementation and improvement of services, through partnerships, in collaboration with professionals and providers.	[39,41,52,53,54,55,57,61,63,64 66,67,68] (N = 13)
7	**Shared responsibility and accountability**	The acknowledgment that multiple actors are responsible and accountable for the quality and outcomes of care, based on collective ownership of actions, goals and objectives, between clients, their families, professionals and providers.	[39,41,52,53,54,56,58,60,62,63,65,68,70] (N = 13)
8	**Continuous**	Services that are consistent, coherent and connected, that address the needs and preferences of clients across their life course.	[39,41,52,53,55,58,59,60,61,68,70,71] (N = 12)
9	**Holistic**	Putting the clients and their needs in the center of the service, whole person oriented, with an eye for physical, social, socio-economical, biomedical, psychological, spiritual and emotional dimensions.	[39,41,52,54,55,57,59,64,66,67,71] (N = 11)
10	**Goal oriented**	Working with clearly described, concrete, measurable, common goals and objectives for clients, their families, professionals and providers.	[39,41,53,58,61,62,63,64,65,66,69] (N = 11)
11	**Personal**	Delivering care by establishing personal contact and relationships, to ensure that services and communication are based on the unique situations of clients and their families.	[39,52,53,59,61,64,66,68,70,71] (N = 10)
12	**Evidence-informed**	Working processes, policies and strategies are guided by evidence-based knowledge, data and information, supported by technology and periodic assessment.	[41,53,54,55,58,59,61,62,62,63,66] (N = 10)
13	**Respectful**	Treating people with respect and dignity, being aware of their experiences, feelings, perceptions, culture and social circumstances.	[41,53,54,56,57,58,63,64,68,71] (N = 10)
14	**Equitable**	Services are accessible and available for all people, and they are all treated equally.	[39,41,52,53,55,58,59,68,70] (N = 9)
15	**Sustainable**	Services are efficient, effective and economically viable, ensuring that they can adapt to evolving environments.	[41,53,54,58,59,60,63,70] (N = 8)
16	**Led by whole-systems thinking**	Taking interrelatedness and interconnectedness into account, realising changes in one part of the system can affect other parts.	[39,41,54,55,57,60,64,69] (N = 8)
17	**Flexible**	Care that is able to change quickly and effectively, to respond to the unique, evolving needs of clients and their families, both in professional teams and organisations.	[54,62,63,64,68,70,71] (N = 7)
18	**Preventative**	Early detection and action for clients and their families that promotes individual and public health.	[41,55,59,66,67,70] (N = 6)
19	**Reciprocal**	Care based on equal, interdependent relationships between clients, their families, professionals and providers, and facilitate cooperative, mutual exchange of knowledge, information and other resources.	[54,63,64,65,69] (N = 5)
20	**Innovative**	Supporting, facilitating and creating space for innovation and future improvements in professional teams and organisations.	[53,61,62,63] (N = 4)
21	**Trustful**	Enabling mutual trust between clients, their families, communities, professionals and organisations, in and across teams.	[54,63,65,66] (N = 4)
22	**Proficient**	Knowledgeable and skilful services are provided by professionals, with a focus on quality.	[52,62,71] (N = 3)
23	**Safe**	Care services that are safe for clients, their families and professionals, including privacy and confidentiality protection.	[52,55,58] (N = 3)

## Discussion

The main objective of this study was to identify the values underpinning integrated care that are evident in the current literature. A systematic literature review was performed which resulted in a set of 23 values and a description of each. Using insights from the articles of Hitlin and Piliavin [30], Schwartz [31] and Rokeach [47] we discuss the interconnectedness of values, the perspectives of different stakeholders on values, and their applicability at different levels and in different contexts. Lastly, the practice and research implications and some methodological considerations are addressed.

### Interconnectedness of values

The list of values presented consists of both values specific to integrated care and values that are more generally related to healthcare delivery. Frequently identified values such as ‘collaborative’ (N = 20), ‘co-ordinated’ (N = 19) and ‘transparent’ (N = 15) could specifically reflect the concept of integrated care. More general values, for example ‘goal oriented’ (N = 11), ‘evidence-informed’ (N = 10), ‘innovative’ (N = 4) and ‘safe’ (N = 3), have been identified less often. Although these values appear to be more generic, they are presumably just as relevant for integrated health services delivery. Thus, the complete set of 23 values consists of a mix of integrated care specific and more generic values with respect to healthcare.

The 23 values presented also seem to embrace themes or concepts that reflect certain goals. In the integrated care literature, these themes are related to the Triple Aim philosophy, in which three main types of goals can be distinguished: client experience, population health and cost-effectiveness [7]. As an example: the values ‘personal’ and ‘respectful’ relate to client experience; ‘preventative’ and ‘equitable’ to population health and ‘sustainable’ to cost-effectiveness. Thus, some of the values may relate to the same overarching themes. Since the Triple Aim goals are interdependent and highly linked to one another, it is arguable that some values could serve as conditions for other values. For example, ‘sustainable’ (related to cost-effectiveness) in relation to ‘continuous’ (experience of care and population health). An integrated care practice that is not sustainable and may not be viable in a certain context is likely to experience problems in delivering continuous care. It would also be difficult to deliver ‘continuous’ care for patients, when care is not ‘comprehensive’. Services cannot consistently address the needs and preferences across the life course of an individual, if some disciplines are not available. So when reviewing our analysis, the values identified would seem to be connected to one another to some extent.

### Stakeholder perspectives and value hierarchies

In integrated care practice, numerous groups of stakeholders are involved, e.g. clients and their families, a variety of care organisations, governmental bodies, professionals, managers and volunteers. When looking at values literature, insights of Hitlin and Piliavin [30], Schwartz [31] and Rokeach [47] suggest that different groups of people distinguish themselves by the relative importance they attach to certain values, also known as their value hierarchies [30,31]. These value hierarchies can help to explain their behaviours and attitudes [31,47]. Other research also supports the premise that different stakeholders can have different perceptions about the same situation. Research by Huber and colleagues illustrates that different stakeholders (patients, public health actors, healthcare providers, citizens, insurers, researchers and policy makers) vary in the importance they attach to various dimensions of health [72]. Other studies show discrepancies between the health state preferences of clients and healthcare professionals [73] and that stakeholders in the same integrated care network may assess the development of their network differently [74].

Assuming that there is a relationship between value hierarchies and behaviours, it is interesting to reflect on the perspectives of stakeholders on the values. For instance, health insurers might attach more importance to values related to costs, such as ‘sustainable’, while clients and professionals might attach more importance to values related to the experience of care, such as ‘trustful’ and ‘respectful’.

When looking at the presented results in this study, the majority of the articles included in the review is written from a researcher’s or expert’s point of view (N = 18). Of a total of 22 included publications, one article elaborates on patient perceptions of integrated care [68] and three articles take the perspectives of health care professionals into account [57,63,65]. It is, therefore, difficult to draw any hard conclusions on the values of professionals or clients.

Considering the insights of Hitlin and Piliavin [30], Schwartz [31], Rokeach [47] and other available knowledge, it is likely that individuals and groups will have different perspectives on the 23 values identified in this study. This is a valuable insight into the explanation of behaviour and collaboration processes in integrated care. More research on these different perspectives, for instance of clients, health care professionals or policymakers, could therefore be interesting.

### Values at different levels and in different contexts

Considering that integrated care is practiced at multiple levels, some of the articles included in our analysis contain a distinction between different levels in the description of their findings [39,56,60,62,63]. Clark and colleagues [56], for example, distinguish individual, team and organisation level, at which the application of a value as ‘respectful’ is described differently: “Develop self and disciplinary knowledge as basis for mutual respect among team members” (individual), “Promote respect, truthtelling, beneficence, and justice in relationships with other team members” (team) and “Respect unique relationship between the team and the patient” (organisation) [56, p. 594]. Another example is Valentijn’s Rainbow Model, that may also be helpful when considering integrated care from a multi-level perspective. This model identifies three levels of integration which are connected, namely the macro (system), meso (organisational, professional) and micro (clinical) levels [39].

Furthermore, in the articles included, the values identified are described on different levels of abstraction. For example, the value ‘holistic’ may be appropriate at the clinical micro-level, where an individual can be viewed holistically taking into account a wide range of factors [59]. This approach should be extended to apply to the meso-level where expertise is shared between professionals from different disciplines [66]. In the articles analysed in this study the value ‘shared accountability and responsibility’ was described at all levels: the macro-level between organisations [70], the meso-level between professionals [52,57,63] and the micro-level between clients and professionals [39]. These examples demonstrate that values can transcend multiple levels in integrated care. This is in line with the insights of Schwartz [31] and Schwartz and Bilsky [50] who state that values transcend specific situations and remain relevant in multiple contexts.

As well as transcending multiple levels, the values identified in this study may also be applicable in multiple contexts. Busetto’s Context + Mechanism + Outcome Model (COMIC model) stresses the importance of taking contexts into account and identifies different categories of contextual factors that can influence integrated care: i.e. innovation, factors with respect to the individual professional and patient, social context, organisational context, health system context and economic, political and legal contexts [8]. In our view, it is important to recognise that studies from seven different countries were included, therefore they probably reflect different economic, political and legal contexts. In this respect, although half of the articles are from the United States (N = 11), the 22 articles cover a broad range of contexts. The value ‘empowering’, for instance, could be interpreted in different ways depending on the different social and political contexts.

Although used differently, the set of values identified for integrated care appears to be applicable and relevant at multiple levels and in multiple contexts. Furthermore, these values are subject to varying interpretation at different levels or in different contexts. By implication, it should be recognised that values can be applied differently in different situations, and that it could be beneficial to distinguish different levels and contexts in any guidance for collaboration in integrated care.

### Practice implications

Since values can help us understand individual behaviour, the values-driven perspective presented in this study can provide insights into the drivers behind the behaviours of the various actors involved in integrated care. A deeper understanding of these underlying mechanisms, can help explain events, dynamics and behaviours in integrated care implementation and delivery. In our view, this can support the further development of integrated care.

Subsequently, values underpinning integrated care could form the basis on which to develop a framework for governance to serve as a guide or steering mechanisms in behaviour, decision-making and evaluation of integrated care. It could be prudent to distinguish different levels and contexts in this respect. At the macro-level, values can help integrated care initiatives in the definition of a collective desirable goal [50], for instance concerning quality, and its evaluation. Values could serve as governance principles for the alignment between organisations in integrated care initiatives and partnerships. For instance by providing a backbone in decision-making processes or leadership behaviour. They can also be used as guiding principles for strategy development and management. At the meso-level, values could also play a role in guiding multidisciplinary care professionals in their daily collaboration with colleagues and other parties involved in integrated care delivery, such as volunteers. At the micro-level, values could serve as professional guiding principles in the delivery of healthcare services. For instance, if ‘co-produced’ is considered to be an important value, professionals could decide to engage clients and their families more. Lastly, a better understanding of the values of clients could facilitate their involvement in integrated care.

### Research implications

As a result of a systematic literature review, this study defines a set of 23 values which are applicable to integrated care. This study also considers the interconnectedness of values, different stakeholder perspectives on values and their applicability to different levels and in different contexts. The findings of this study broaden the existing body of knowledge on integrated care, by combining integrated care theory and values theory. There have been no systematic studies about values in integrated care published until now and this study therefore bridges this gap. The set of values presented can be seen as a potential basis for a values-driven approach to integrated care. From an academic perspective, the values identified can also contribute to the clarification and definition of integrated care as a concept.

Future research could examine the differences in value hierarchies of clients, professionals and decision-makers and the potential effect of different perspectives on integrated care. It would also be interesting to investigate the applicability of the set of values to different levels of integrated care and in different contexts. Another avenue of research could be to further determine which values are specific to integrated care, and to what extent the set of underlying values is suitable for use as a framework for governance or a steering mechanism in practice. Lastly, it would be interesting to study the extent to which values can be used to align collaboration in integrated care and their use in helping professionals to make decisions.

### Strengths and limitations

One of the strengths of this study is the systematic theory-driven approach to the research. It adds value by expanding the theoretical body of knowledge on integrated care described in current literature which includes definitions, conceptual models and interventions. Recent academic literature has been identified, assessed and analysed using a defined method, executed by three independent researchers and a supervisor. A limitation of the study is that the 22 articles analysed are predominantly written from an expert or academic perspective (N = 18) and to a lesser extent from a client (N = 1) or professional (N = 3). Therefore, we cannot draw any hard conclusions about the perspectives of professionals, clients or their families with respect to the values of integrated care. This could be a topic for further research.

## Conclusion

For better understanding of behaviour, decision-making and collaboration in integrated care, more insight into the underlying values of integrated care is needed. Although more attention is being paid to values in integrated care, this topic has not been systematically studied until now. This study bridges this gap by applying values theory to the integrated care literature. A set of 23 values and a description of each are presented. This set of values is proposed as a potential basis for a values-driven approach to integrated care. The results of this research can be used as a basis for the guidance of collaboration and governance processes in integrated care and add to conceptual knowledge and theory building of integrated care.

## Additional Files

The additional files for this article can be found as follows:

10.5334/ijic.4172.s1 Appendix 1 Search strategies.

10.5334/ijic.4172.s2 Appendix 2 Articles included in the systematic review.

10.5334/ijic.4172.s3 Appendix 3 Dataset of the systematic review.
