# Supplementary material for: Values of Integrated Care: A Systematic Review
Source: Int J Integr Care. 2018 Nov 15;18(4):9. doi: 10.5334/ijic.4172 (PMC6251066; doi:10.5334/ijic.4172)
Supplement: Appendix 1 — Search strategies. [file ijic-18-4-4172-s1.pdf]

Appendix 1: Search strategies.

Table 3: Search strategy 1.

[Title] values OR principles OR fundament* OR key beliefs OR philosophy OR theor* OR concept*	AND	[Title] person centered OR person centred OR patient centered OR patient centred OR people centered OR people centred OR client centered OR client centred OR integrat* OR coordination OR interprofessional OR interorganizational OR interorganisational OR interdisciplinary OR collaboration OR cooperation	AND	[Title] care OR healthcare OR health services OR health delivery OR health service delivery	NOT	[Title] laboratory OR dental OR oncology OR surgery OR surgical OR pharmacy OR catholic OR radiology OR biomedical OR software OR electronic OR information system OR algorithm

Table 4: Search strategy 2.

[Title/Abstract] model OR models OR framework OR frameworks	AND	[Title/Abstract] person centered OR person centred OR patient centered OR patient centred OR people centered OR people centred OR client centered OR client centred OR integrat* OR coordination OR interprofessional OR interorganizational OR interorganisational OR interdisciplinary OR collaboration OR cooperation	AND	[Title] care OR healthcare OR health services OR health delivery OR health service delivery	NOT	[Title] laboratory OR dental OR oncology OR surgery OR surgical OR pharmacy OR catholic OR radiology OR biomedical OR software OR electronic OR information system OR algorithm

* [MeSH Major Topic] Models, Organizational
