# Supplementary material for: Values of Integrated Care: A Systematic Review
Source: Int J Integr Care. 2018 Nov 15;18(4):9. doi: 10.5334/ijic.4172 (PMC6251066; doi:10.5334/ijic.4172)
Supplement: Appendix 2 — Articles included in the systematic review. [file ijic-18-4-4172-s2.pdf]

Appendix 2: Articles included in the systematic review.

Table 5: Articles included in the systematic review.

Ref.	Author	Year	Title	Scope of the article	Methods	Identified values (N)
[52]	Baird, M., Blount, A., Brungardt, S., Dickinson, P., Dietrich, A., Epperly, T., … others.	2014	Joint principles: integrating behavioral health care into the patient-centered medical home.	The representatives from six American Family Medicine Organisations developed a set of Joint Principles for Integrating Behavioral Health Care into Patient-Centered Medical Home.	Experiences of experts, focus groups	Collaborative, Co-ordinated, Transparent, Comprehensive, Co-produced, Shared accountability and responsibility, Continuous, Holistic, Personal, Equitable, Proficient, Safe (N=12)
[53]	Bodenheimer, T., Ghorob, A., Willard-Grace, R., & Grumbach, K.	2014	The 10 building blocks of high-performing primary care.	Ten building blocks of high performing primary care (in coordination with other services) were developed by the review of literature, site visits and practical experience of the authors.	Literature review, experiences of experts, site visits	Collaborative, Co-ordinated, Empowering, Comprehensive, Co-produced, Shared accountability and responsibility, Continuous, Goal oriented, Personal, Evidence-informed, Respectful, Equitable, Sustainable, Innovative (N=14)
[54]	Butt, G., Markle-Reid, M., & Browne, G.	2008	Interprofessional partnerships in chronic illness care: a conceptual model for measuring partnership effectiveness	This paper presents a conceptual model for Interprofessional Health and Social Service Partnerships (IHSSP), derived from a systematic literature review.	Literature review	Collaborative, Co-ordinated, Transparent, Comprehensive, Co-produced, Shared accountability and responsibility, Holistic, Evidence-informed, Respectful, Sustainable, Led by whole-systems thinking, Flexible, Reciprocal, Trustful (N=14)
[55]	Callahan, C. M., Boustani, M.	2011	Implementing dementia care models in primary care settings:	This article describes the experiences in the implementation of a collaborative	Experiences of experts	Collaborative, Co-ordinated, Empowering, Co-produced, Continuous,

	A., Weiner, M., Beck, R. A., Livin, L. R., Kellams, J. J., … Hendrie, H. C.		The Aging Brain Care Medical Home.	care model for dementia and depression.		Holistic, Evidence-informed, Equitable, Led by whole-systems thinking, Preventative, Safe (N=11)
[56]	Clark, P. G., Cott, C., & Drinka, T. J.	2007	Theory and practice in interprofessional ethics: A framework for understanding ethical issues in health care teams.	This article presents a framework for interprofessional teamwork in healthcare practice, based on a literature review and the experiences of experts.	Literature review, experiences of experts	Collaborative, Shared accountability and responsibility, Respectful (N=3)
[57]	Craig, S. L., Betancourt, I., & Muskat, B. Social Work & Healthcare	2015	Thinking big, supporting families and enabling coping: The value of social work in patient and family centered health care.	In this paper the value of social work in healthcare practice is described from the perspectives of health social workers.	Interviews, focus groups,	Collaborative, Transparent, Empowering, Co-produced, Holistic, Respectful, Led by whole-systems thinking (N=7)
[41]	Ferrer, L., & Goodwin, N.	2014	What are the principles that underpin integrated care?	Ferrer and Goodwin present 16 core principles of integrated care, by reflecting on the views and comments from international stakeholders involved in the development of the WHO Global Strategy on People-Centred and Integrated Health Service.	Experiences of experts, reflection on strategy development	Collaborative, Co-ordinated, Empowering, Comprehensive, Co-produced, Shared accountability and responsibility, Continuous, Holistic, Goal oriented, Evidence-informed, Respectful, Equitable, Sustainable, Led by whole-systems thinking, Preventative (N=15)
[59]	Johnson	2009	Health care	This article	Review/Co	Collaborative, Co-

	, C.		transitions: a review of integrated, integrative, and integration concepts.	presents a short review on the concepts integrated, integrative and integration in health care transitions.	ncept analysis	ordinated, Transparent, Empowering, Comprehensive, Continuous, Holistic, Personal, Evidence-informed, Equitable, Sustainable, Preventative (N=12)
[60]	Kodner, D. L.	2008	All together now: a conceptual exploration of integrated care.	This article explores definitions, concepts, logics and methods found in health system and service integration.	Review/Concept analysis	Collaborative, Co-ordinated, Empowering, Comprehensive, Shared accountability and responsibility, Continuous, Sustainable, Led by whole-systems thinking (N=8)
[70]	Lega, F.	2007	Organisational design for health integrated delivery systems: theory and practice.	This paper presents an organisational design for health integrated delivery systems, based on an extensive literature review.	Literature review, experiences from action research	Co-ordinated, Comprehensive, Shared accountability and responsibility, Continuous, Personal, Equitable, Sustainable, Flexible, Preventative (N=9)
[61]	Minkman, M., Ahaus, K., Fabbricotti, I., Nabitz, U., & Huijsman, R.	2008	A quality management model for integrated care: results of a Delphi and Concept Mapping study.	This study presents 89 elements of integrated care, grouped into nine core clusters. The elements and clusters are identified by experts, taking part in a Delphi Study and Concept Mapping.	Delphi Study, concept mapping	Collaborative, Co-ordinated, Transparent, Empowering, Co-produced, Continuous, Goal oriented, Personal, Evidence-informed, Innovative (N=10)
[62]	Mulvale, G., Embrett, M., & Razavi, S. D.	2016	‘Gearing Up’ to improve interprofessional collaboration in primary care: a systematic review and	A systematic review of 25 years of peer-review literature was conducted to develop a conceptual framework for interprofessional	Literature review	Collaborative, Transparent, Shared accountability and responsibility, Goal oriented, Evidence-informed, Flexible, Innovative, Proficient (N=8)

			conceptual framework	collaboration in primay care.		
[63]	Poochikian-Sarkissian, S., Hunter, J., Tully, S., Lazar, N. M., Sabo, K., & Cursio, C.	2008	Developing an innovative care delivery model: Interprofessional practice teams.	This paper presents an innovative care delivery model, by doing focus groups and surveys with members of interprofessional practice teams.	Focus groups, surveys	Collaborative, Co-ordinated, Transparent, Empowering, Comprehensive, Co-produced, Shared accountability and responsibility, Goal oriented, Evidence-informed, Respectful, Sustainable, Flexible, Reciprocal, Innovative, Trustful (N=15)
[64]	Prokop, J.	2016	Care coordination strategies in reforming health care: a concept analysis.	This article describes a concept analysis of care coordination: definitions, attributes and antecedents.	Review/Concept analysis	Collaborative, Co-ordinated, Transparent, Empowering, Comprehensive, Co-produced, Holistic, Goal oriented, Personal, Respectful, Led by whole-systems thinking, Flexible, Reciprocal (N=13)
[71]	Radwin, L. E., Castonguay, D., Keenan, C. B., & Hermann, C.	2016	An expanded theoretical framework of care coordination across transitions in care settings.	This study builds upon three existing models, in order to build an expanded theoretical framework of care coordination across transitions in care settings.	Review/Concept analysis	Co-ordinated, Transparent, Continuous, Holistic, Personal, Respectful, Flexible, Proficient (N=8)
[65]	Shaw, J., Kearney, C., Glenns, B., & McKay, S.	2016	Interprofessional team building in the palliative home care setting: Use of a conceptual framework to inform a pilot evaluation.	This paper presents a conceptual framework on interprofessional teams and an evaluation of interprofessional teams in palliative care by doing interviews.	Interviews	Collaborative, Co-ordinated, Transparent, Shared accountability and responsibility, Goal oriented, Reciprocal, Trustful (N=7)

[66]	Suter, P., Hennessey, B., Harrison, G., Fagan, M., Norman, B., & Suter, W. N.	2008	Home-Based Chronic Care: An Expanded Integrative Model for Home Health Professionals.	This article builds upon Wagner's Chronic Care Model and integrates salient theories from fields beyond medicine, resulting in an expanded Home-Based Chronic Care Model (HBCCM).	Review/Concept analysis	Collaborative, Co-ordinated, Transparent, Empowering, Co-produced, Holistic, Goal oriented, Personal, Evidence-informed, Preventative, Trustful (N=11)
[67]	Thornton, L.	2013	Essentials of Integrative Health Care: Fundamental Principles for Caring& Healing.	This study presents a set of essential Components of Integrative Care, by reviewing existing models and practice experience.	Review/Concept analysis	Collaborative, Co-ordinated, Transparent, Empowering, Comprehensive, Co-produced, Holistic, Preventative (N=8)
[39]	Valentijn, P., Schepman, S. M., Opheij, W., & Bruijnzeels, M. A	2013	Understanding integrated care: a comprehensive conceptual framework based on the integrative functions of primary care	This article presents a conceptual framework on the integrative functions of primary care, based on a literature review and focus groups with experts.	Literature review, focus groups	Collaborative, Co-ordinated, Empowering, Comprehensive, Co-produced, Shared accountability and responsibility, Continuous, Holistic, Goal oriented, Personal, Equitable, Led by whole-systems thinking (N=12)
[58]	Van Houdt, S., Heyrman, J., Vanhaecht, K., Sermeus, W., & De Lepeleire, J.	2013	An in-depth analysis of theoretical frameworks for the study of care coordination	This study describes an update framework for care coordination, developed by performing a literature review on existing theoretical frameworks.	Literature review	Collaborative, Co-ordinated, Transparent, Comprehensive, Shared accountability and responsibility, Continuous, Goal oriented, Evidence-informed, Respectful, Equitable, Sustainable, Safe (N=12)
[68]	Walker, K. O., Labat, A., Choi,	2013	Patient perceptions of integrated care: confused by	This paper presents the results of seven focus groups on perceptions of the concept of	Focus groups	Collaborative, Co-ordinated, Transparent, Empowering, Comprehensive, Co-

	J., Schmittdiel, J., Stewart, A. L., & Grumbach, K.		the term, clear on the concept.	integrated care with 44 patients.		produced, Shared accountability and responsibility, Continuous, Personal, Respectful, Equitable, Flexible (N=12)
[69]	Winge, M., Johansson, L.-\AAke, Nyström, M., Lindh-Waterworth, E., & Wangler, B.	2010	Need for a New Care Model- Getting to Grips with Collaborative Home Care.	This paper discusses that patients are increasingly treated in their homes by a set of collaborating organisations, and therefore underlines the need for a new collaborative care model.	Review/Concept analysis	Collaborative, Co-ordinated, Transparent, Goal oriented, Led by whole-systems thinking, Reciprocal (N=6)
